# C-phycocyanin as a highly attractive model system in protein crystallography: unique crystallization properties and packing-diversity screening

**DOI:** 10.1107/S2059798320016071

**Published:** 2021-01-26

**Authors:** Iosifina Sarrou, Christian G. Feiler, Sven Falke, Nolan Peard, Oleksandr Yefanov, Henry Chapman

**Affiliations:** a Centre for Free-Electron Laser Science, DESY, Notkestrasse 85, 22607 Hamburg, Germany; b Helmholtz-Zentrum Berlin für Materialien und Energie, Albert-Einstein-Strasse 15, 12489 Berlin, Germany; c Laboratory for Structural Biology of Infection and Inflammation, Universität Hamburg, Notkestrasse 85, 22607 Hamburg, Germany; d Hamburg Centre for Ultrafast Imaging, Universität Hamburg, Luruper Chaussee 149, 22607 Hamburg, Germany; e Department of Physics, Massachusetts Institute of Technology, 77 Massachusetts Avenue, Cambridge, MA 02139, USA; f Department of Physics, Universität Hamburg, Luruper Chaussee 149, 22607 Hamburg, Germany

**Keywords:** C-phycocyanin, crystal packing, model system, precipitant, crystallization determinants

## Abstract

The unique crystallization properties of the antenna protein C-phycocyanin (C-PC) from the thermophilic cyanobacterium *Thermosynechococcus elongatus* are reported and discussed. C-PC crystallizes in hundreds of significantly different conditions within a broad pH range and in the presence of a wide variety of precipitants and additives. Remarkably, the crystal dimensions vary from a few micrometres, as used in serial crystallography, to several hundred micrometres, with a very diverse crystal morphology. More than 100 unique single-crystal X-ray diffraction data sets were collected from randomly selected crystals and analysed. The addition of small-molecule additives revealed three new crystal packings of C-PC, which are discussed in detail. The high propensity of this protein to crystallize, combined with its natural blue colour and its fluorescence characteristics, make it an excellent candidate as a superior and highly adaptable model system in crystallography. C-PC can be used in technical and methods development approaches for X-ray and neutron diffraction techniques, and as a system for comprehending the fundamental principles of protein crystallography.

## Abstract 2

C-phycocyanin, a photosynthetic antenna protein from the cyanobacterium *Thermosynechococcus elongatus*, was crystallized in hundreds of different conditions, producing protein crystals with various sizes and morphologies. Among the many diffraction data sets that were collected, high-resolution X-ray structures with novel symmetries were solved and are discussed with the aim of providing a highly adaptable experimental model system.

## 1. Introduction   

Protein X-ray crystallographic techniques have been used extensively to determine static macromolecular structures and also to analyse protein dynamics (Liebschner, 2018 ▸; Spence, 2017 ▸). Hence, high-quality protein crystals are necessary to achieve electron-density maps of high resolution and high confidence, which allow the study of molecular mechanisms and of the function and interactions of biomacromolecules (Liebschner, 2018 ▸; Chayen, 2009 ▸).

Protein crystallization is a multi-parametric process and depends on several factors, including protein concentration, sample purity, temperature, pH value, precipitant, buffers, additives, detergents, force fields and pressure, which can be visualized in multi-dimensional phase diagrams (Chayen *et al.*, 1992 ▸; Chayen, 2009 ▸; Rupp, 2010 ▸; McPherson & Cudney, 2006 ▸). Protein crystal nucleation is described as a multi-step process with the early formation of a dense liquid protein phase, which precedes the growth of a rigid nucleus (Sauter *et al.*, 2015 ▸).

The most popular approach to initial crystallization trials applies sparse-matrix screens of 96 different conditions at a time, which are subsequently refined according to the successful hits (Chayen, 2009 ▸; Rupp, 2010 ▸). However, it is challenging to identify those crystallization conditions that yield high-quality diffracting crystals (Chayen & Saridakis, 2008 ▸). It is not feasible to make a prediction, based on the chemical and physical properties of a protein, of the conditions required to crystallize it, even with efforts to monitor and actively influence the crystallization process (Falke & Betzel, 2019 ▸). Changes in a single experimental parameter can simultaneously affect several aspects of a crystallization experiment (Chayen, 2009 ▸). Many efforts, over decades, have aimed at improving the success of crystallization experiments and the resulting crystal size (Rupp, 2010 ▸; McPherson & Cudney, 2006 ▸). However, the success of crystallization methods widely depends on trial and error (DePristo *et al.*, 2004 ▸; Groot *et al.*, 1998 ▸).

Moreover, methods development in macromolecular X-ray crystallography often depends upon the use of easy-to-handle proteins that are highly stable and available in bulk amounts, such as insulin, proteinase K and hen egg-white lysozyme. The crystallization abilities of the latter protein have been well studied, involving different space groups, crystal morphologies and sizes for the development of multiple applications (Panjikar *et al.*, 2015 ▸; Meents *et al.*, 2017 ▸; Haas, 2020 ▸).

This study focuses on examining the robust crystallization of C-phycocyanin (C-PC), which is almost independent of the composition of the crystallization solution, and investigating the resulting crystal morphology, molecular packing and crystal quality. Several crystal structures of the cyanobacterial antenna protein C-PC (Nield *et al.*, 2003 ▸; Adir *et al.*, 2002 ▸) have already been reported and information on selected C-PC structures from various cyanobacteria is shown in Supplementary Table S1.

This study demonstrates that C-PC from *Thermosynecho­coccus elongatus*, purified using a highly efficient one-column purification protocol, can be crystallized using hundreds of diverse crystallization solutions in numerous crystal symmetries at different pH values and with additives. Various crystal morphologies can be recognized, from which several dozen high-resolution X-ray diffraction data sets have been collected. Different crystal packings were observed when small drug-like molecules were present in the crystallization solutions. An in-depth analysis of these molecular assemblies will be helpful in comprehending the uncommon crystallization behaviour of native C-PC.

The antenna protein C-PC appears to be an essential sample for methods development in protein crystallography. In combination with their optical properties, blue colour and strong fluorescence, C-PC crystals may inspire new applications and studies in biomolecular crystallography.

## 2. Materials and methods   

### 2.1. Purification, characterization in solution and crystallization of C-PC   

C-PC from *T. elongatus* was isolated based on a protocol previously described elsewhere (Nield *et al.*, 2003 ▸) with an additional chromatography step using a DEAE chromatography column equilibrated with 20 m*M* MES pH 6.5 or 20 m*M* Tris pH 8.0 (depending on the downstream crystallization experiment) and mounted on an ÄKTA purifier (GE Healthcare, USA). Every litre of cell culture produces 1 g of wet cells, resulting in approximately 8 mg purified C-PC.

When studied at pH 4.0, the protein was dialyzed against 20 m*M* sodium acetate, 100 m*M* NaCl.

CD spectroscopy experiments were performed to verify the overall folding and secondary-structure composition of C-PC in different buffers using a Jasco J-810 spectrometer (Jasco, UK). Spectra of pure C-PC (0.1 mg ml^−1^) were recorded in the far-UV wavelength range between 195 and 240 nm at 20°C using a 1 mm path-length quartz cell with a scanning speed of 100 nm min^−1^. The obtained spectra provide a fingerprint of the secondary-structure composition of C-PC. Ellipticity values were scaled and provided as the mean molar ellipticity (MME). Ten spectra were averaged.

The dispersity and particle-size distribution of the protein in solutions at different pH values was verified via infrared dynamic light scattering (IR-DLS) using a DynaPro NanoStar instrument (Wyatt Instruments, USA) equipped with a 785 nm wavelength laser. C-PC naturally has a strong absorption maximum at approximately 600 nm and fluorescence at about 640 nm. The optical properties of C-PC are taken into account when the protein is studied in solution and the crystalline form.

Prior to crystallization, C-PC was dialyzed against the respective chromatography buffer supplemented with 100 m*M* NaCl. The protein buffer concentration was limited to 20 m*M* in order to maximize the effect of extreme pH values of the crystallization solutions after mixing.

The protein was concentrated using Amicon Centricon YM-10 centrifugal filters at 1500*g* to a final concentration of 15 mg ml^−1^.

The crystallization experiments were set up manually in 96-well plates (MRC 2-well; Jena Bioscience) using the commercially available screens JCSG-*plus*, Morpheus, Morpheus 3, Pi-Minimal, PACT, SG1, PGA and Midas from Molecular Dimensions and Jena Bioscience. In each sitting-drop vapour-diffusion experiment, 1 µl protein solution was mixed with 1 µl reservoir solution and incubated against 50 µl reservoir solution. Crystallization plates were incubated at 22°C and automatically imaged by second-order nonlinear imaging of chiral crystals (SONICC; Formulatrix, Bedford, Massachusetts, USA).

The presence of crystalline material (>1 µm) was verified in each crystallization drop using SONICC. This second-harmonic generation (SHG) imaging relies on a nonlinear optical process of frequency doubling in chiral non-centrosymmetric crystals to provide information on the crystallinity (Boyd, 2008 ▸). The UV-TPEF (ultraviolet two-photon excited fluorescence) method is analogous to classical UV fluorescence and generates images based on the fluorescence of UV-excited aromatic amino acids.

All crystals obtained grew to full size within two days or less, with various sizes and different shapes. Unless the crystallization condition contained a cryoprotectant, crystals were briefly washed with a cryoprotectant solution containing the mother liquor supplemented with 25% PEG 400 before flash-cooling in liquid nitrogen.

### 2.2. Data collection   

Diffraction data were collected from single crystals on beamline P11 at the PETRA III electron-storage ring, DESY, Hamburg, using a PILATUS3 S 6M detector. All data were collected as non-overlapping 0.1° oscillation images, indexed and integrated with *XDSAPP* (Sparta *et al.*, 2016 ▸), and scaled with *AIMLESS* from the *CCP*4 suite (Winn *et al.*, 2011 ▸). A statistically significant value for CC_1/2_ (Karplus & Diederichs, 2012 ▸) in the highest resolution shell was chosen as a cutoff criterion respecting the completeness of the data. Indexing parameters are summarized in Supplementary Tables S2 and S3. Collected raw diffraction images are publicly available via https://proteindiffraction.org/.

### 2.3. Structure determination and refinement   

The crystal structures of C-PC were determined by molecular replacement with *phenix.phaser* (Liebschner *et al.*, 2019 ▸) using a heterodimer extracted from PDB entry 1jbo (Nield *et al.*, 2003 ▸) as a search model. Iterative automated refinement was carried out with *phenix.refine* (Liebschner *et al.*, 2019 ▸), and manual adjustments and model optimization were performed by hand in *Coot* (Emsley *et al.*, 2010 ▸). Structural coordinates have been deposited in the Protein Data Bank with accession codes 6yyj (space group *P*2_1_2_1_2), 6yq8 (space group *P*6_3_, larger unit cell), 6yqg (space group *P*6_3_, smaller unit cell) and 6ypq (space group *R*32). Data-collection and refinement statistics are also summarized in Tables 1 ▸ and 2 ▸.

## 3. Results and discussion   

### 3.1. Purification of C-PC and characterization in solution   

**Figure 1:**
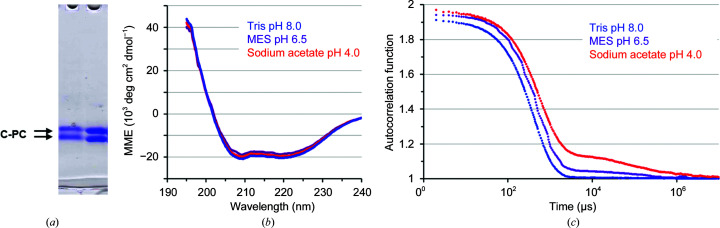
Characterization of C-PC sample solutions prior to crystallization. (*a*) SDS–PAGE (12% acrylamide) analysis of the chromatographic fractions containing pure C-PC used in crystallization trials. (*b*) Averaged spectra of C-PC obtained by CD spectroscopy buffered at pH 8.0 (20 m*M* Tris; blue), pH 6.5 (20 m*M* MES; purple) and pH 4.0 (20 m*M* acetate; red), indicating a nearly identical secondary structure in all three buffers. (*c*) Autocorrelation functions of C-PC depending on the delay time as obtained by IR-DLS indicate an increased degree of C-PC solution polydispersity and additional larger particles at pH 4.0 compared with pH 8.0 and pH 6.5.

Before the crystallization experiments, C-PC was purified to homogeneity and characterized in solution using CD spectroscopy to verify the folding and DLS to investigate the solution dispersity (Fig. 1 ▸). As observed using IR-DLS measurements, prior to crystallization, with C-PC at pH 4.0, 6.5 and 8.0, the hydrodynamic radius is significantly increased at pH 4.0 with increased polydispersity. C-PC in solution at pH 8.0, 6.5 and 4.0 showed hydrodynamic radii of 4.2 nm (12% polydispersity), 4.8 nm (32% polydispersity) and 6.3 nm (40% polydispersity), respectively.

Interestingly, after 72 h, in the protein solution at pH 4.0 particles with lattice order and diameters in the range 4–20 µm appeared; this was not the case for the protein at pH 6.5 and pH 8.0. These micrometre-sized particles were indeed identified as crystalline material. Diffraction data of self-assembled crystals were collected by a serial crystallography approach using a porous polyimide support (Feiler *et al.*, 2019 ▸; Supplementary Figs. S1*c* and S1*d*), the results will be published elsewhere in more detail. Therefore, we conclude that lower pH values promote the crystallization of C-PC via auto-assembly. The higher percentage of crystallization conditions providing crystals when the protein was buffered at pH 6.5 instead of pH 8.0 would be consistent with these results. The particular reasons for the self-assembly of C-PC towards crystalline particles at lower pH are still under investigation and might be connected to the *in vivo* function of C-PC, since phycobilosomes are naturally organized into rods attached to the thylakoid membrane in cyanobacteria and algae (Blankenship, 2015 ▸).

Based on these results, crystallization setups were focused on using C-PC buffered at pH 8.0 and pH 6.5.

### 3.2. Crystallization experiments   

The crystallization plates were automatically imaged at 22°C and two types of image data were recorded: visible and SHG (Supplementary Figs. S3 and S4). A numerical summary of the imaging results depending on the screen is shown in Table 3 ▸. The values refer to the total number of conditions with visible crystals or microcrystals which showed SHG signal. To avoid false positives, plates were imaged by UV-TPEF to confirm that the SHG-positive crystals are indeed protein crystals, especially in the case of microcrystals (Supplementary Fig. S2). Since the laser illumination for the imaging causes damage to the protein crystals owing to local heating, the exposure was limited to the default value in order to proceed to X-ray single-crystal experiments.

C-PC in MES buffer pH 6.5 was screened using eight commercially available screens (Table 3 ▸), *i.e.* testing a total number of 786 different crystallization conditions. After 48 h, crystals could be detected in 724 crystallization conditions covering 92% of all conditions tested. For comparison, C-PC in Tris buffer pH 8.0 was screened against four commercial screens (Table 3 ▸), *i.e.* 384 individual conditions were tested. After two days, the plates were inspected and crystals could be detected in 291 conditions that were cross-verified with the SHG signal. All of the imaging results under visible light and SHG of the 96-well plates are shown in Supplementary Figs. S3 and S4.

**Figure 2:**
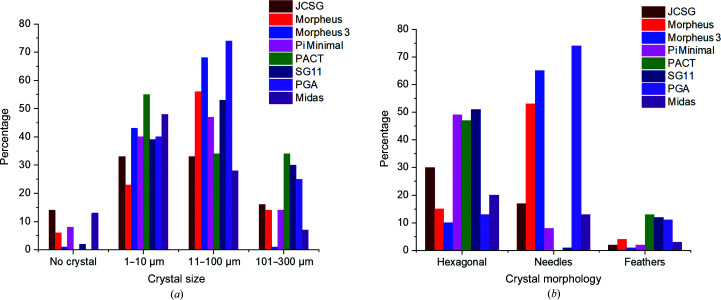
(*a*) The statistics of crystal size as they appear under different conditions. Naturally, crystal sizes may vary within one crystallization drop; the results are determined based on the majority of crystals. In some cases, where the crystals have two distinct representative size regimes, both are included; see the examples in Supplementary Fig. S2. (*b*) Three categories of crystal morphology in the C-PC crystallization experiments utilizing eight different screens as shown in Table 3 ▸, with C-PC in MES buffer at pH 6.5. Please note that the morphologies are reported for crystals larger than 10 µm.

In total, this experiment exhibited protein crystals in more than 1000 different conditions, which appeared in different morphologies and sizes. The details of the statistics for the eight screens with C-PC at pH 6.5 are shown in Fig. 2 ▸. While most of the conditions foster the formation of crystals in general, the space group, maximum resolution and other parameters depend on the individual compositions of the crystallization solution. Furthermore, the screens show large differences in the morphologies and sizes of the crystals. For example, in the PACT screen, which contains PEG in all conditions, there is a majority of hits with hexagonal crystals over hits with needle- or feather-like shapes. However, the different crystal morphologies could neither be correlated with the pH value of the crystallization condition nor with the precipitant. It has not been possible to correlate a specific chemical component with a specific morphology, as attempted in other cases (He *et al.*, 2020 ▸), as several widely different conditions result in the same morphology. On the other hand, one specific crystallization solution (*e.g.* PGA condition B9, Midas condition C6) can also result in a mixture of crystals with significantly different morphology in the same droplet but, as far as we analyzed, with the same space group and molecular packing. Therefore, in contrast to other cases (Frey *et al.*, 1991 ▸), the morphology also does not seem to indicate an individual space group and packing. For some conditions, however, we saw that crystals with different morphologies appeared in the same droplet after significantly different incubation times. This might lead to the very general assumption that the morphology is determined by a combination of the initial crystallization solution composition, which determines, for example, a specific second virial coefficient, and the growth speed, which is affected by the local protein concentration at a specific time of incubation.

**Figure 3:**
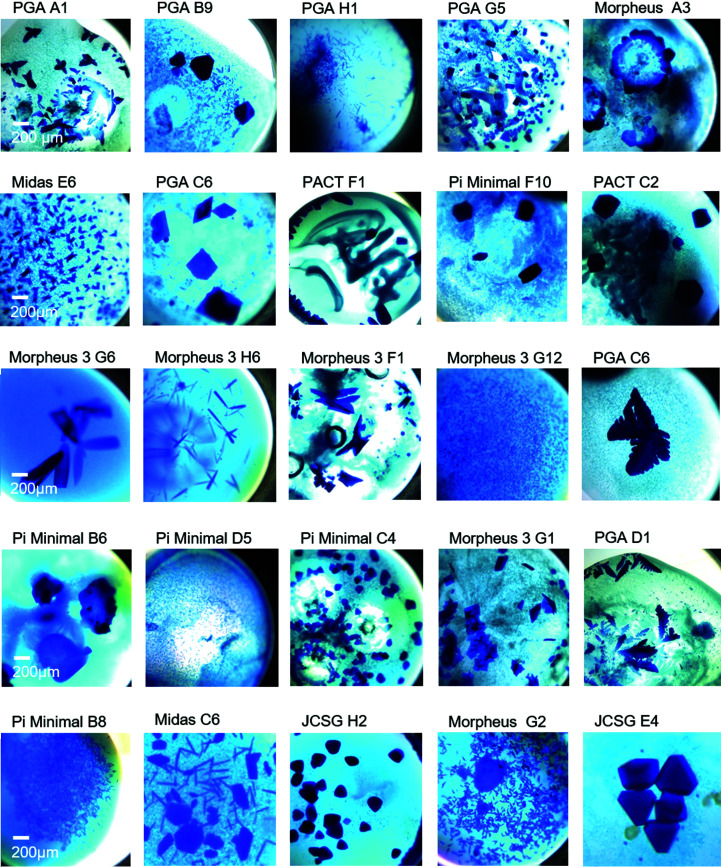
C-PC crystals predominantly appear in three morphologies, hexagonal, needle-like and feather-like, as shown in Fig. 2 ▸. Different crystallization screens favour certain combinations of morphologies and sizes, but there is no direct correlation of the precipitant, pH value or additives that results in certain morphologies.

These unique properties of the crystal formation of C-PC will provide valuable information for future studies of protein crystallogenesis (Lorber, 2005 ▸). It is worth mentioning that similar morphologies with size variations and a dependency on the screen were observed in the trays where the protein was purified at pH 8.0 (Supplementary Fig. S5). Examples portraying the different crystal morphologies observed at 20°C are shown in Fig. 3 ▸.

### 3.3. Data collection   

A few hundred crystals with sizes larger than 70 µm were picked and cryocooled in liquid nitrogen after incubation for a few seconds in a cryoprotectant solution that consisted of the mother liquor supplemented with cryoprotectant when necessary. The crystals were selected from 12 screens, as shown in Table 3 ▸. We checked more than 200 different crystals for diffraction. Table 4 ▸ contains examples of the diverse crystallization conditions which resulted in crystals that diffracted to high resolution. With various combinations of precipitants and pH values, C-PC produces crystals with the same symmetry. Table 5 ▸ summarizes the properties of 118 individually collected data sets. The outcome of the data-collection analysis is presented in Supplementary Tables S2 and S3.

It is worth mentioning that 41 individual crystals diffracted to a resolution of higher than 1.2 Å, which improves the highest maximum resolution of a cyanobacterial C-phycocyanin structure reported to date, which is 1.35 Å (PDB entry 3o18; David *et al.*, 2011 ▸). More than 70% of all data collected extended to a resolution of better than 1.5 Å, and 95% extended to better than 2 Å resolution (Supplementary Fig. S6). This outcome is very likely to be owing to the high purity of C-PC produced using the modified purification method (see Section 2 and Fig. 1 ▸).

Analysis of these results shows that C-PC assembly is not affected by the crystallization conditions. The protein crystallizes in a particular space group independent of the pH value or the precipitants, whether for example high salt, PEG or ethanol are present (examples are provided in Table 4 ▸ and Supplementary Tables S2 and S3). This effect was previously concealed and, remarkably, the tendency of C-PC to preferentially crystallize in space group *R*32 is unexpected. Compared with well characterized crystal model systems, such as hen egg-white lysozyme, for which a vast number of symmetries have been reported, C-PC prefers to crystallize with *R*32 symmetry. As mentioned earlier, the purified protein can self-assemble into nanosized structures in solution. This effect is very likely to promote crystal nucleation and to act as a seed during crystal growth. Since phycobilosomes are naturally organized in the cells (Blankenship, 2015 ▸), the *in vivo* C-PC assembly exhibits a tricylindrical core, from which six rods composed of three PC hexamers radiate, in order to assemble superior rigid antenna-like structures to expand light harvesting (Wang & Moerner, 2015 ▸).

### 3.4. Crystal packing and symmetries   

**Figure 4:**
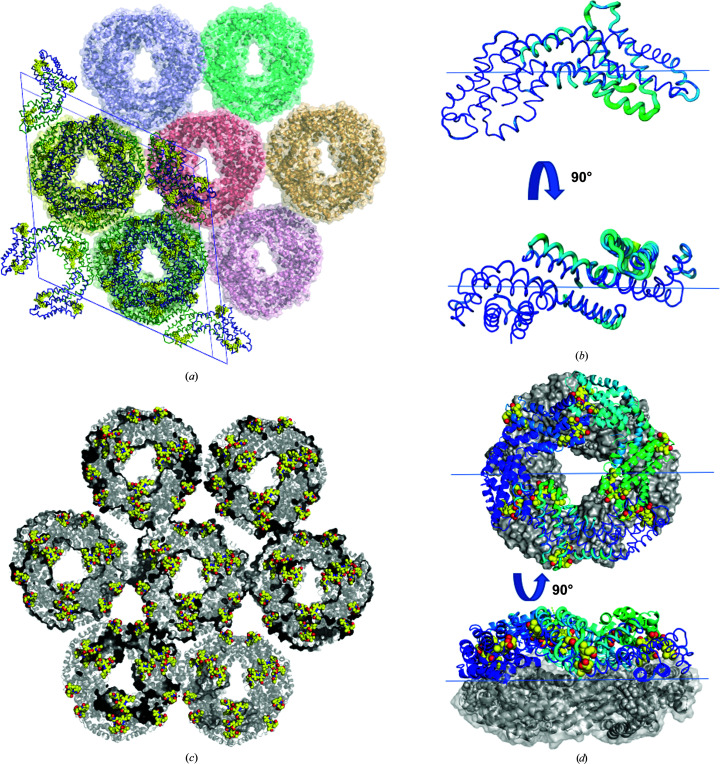
Crystal packing and structural details of C-PC crystallized in the rhombohedral space group *R*32 (data set 10, Supplementary Table S3). (*a*) The unit-cell content is depicted as an overlay in the crystal-packing context. (*b*) *B*-factor plot as a function of position in the individual heterodimeric C-PC molecules. (*c*) Each heterodimeric C-PC molecule harbours three attached phycocyanobilin ligands (yellow). (*d*) The dodecameric ring structure with the top ring in cartoon representation and the bottom ring as a surface (grey).

**Figure 5:**
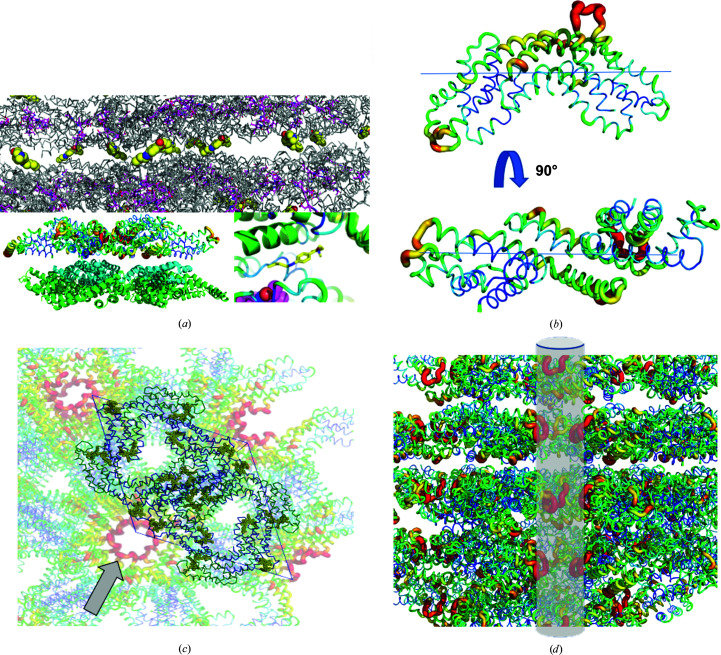
C-PC in the hexagonal space group *P*6_3_ (data set 42, Supplementary Table S2). (*a*) Crystal contacts and the layered hexameric ring structure (coloured grey) are mediated by the additive molecule tetracaine (coloured yellow). (*b*) The *B* factors are plotted onto the heterodimeric structure. (*c*) The unit-cell content is superposed onto the crystal packing coloured according to *B* factors. The arrow points to the small pore flanked by the loop region binding one of the cofactors, coloured dark yellow. (*d*) The tunnel spans across the crystal. Individual layers are coloured according to the *B* factors and the tunnel is depicted in light grey.

Amongst the diffraction data sets that were collected and analysed, there are distinct variations in the crystal packing and the symmetry. As summarized in Table 5 ▸, for the majority of the data that were collected and analysed, C-PC crystallizes in high-symmetry space groups. More precisely, there are four different space groups. The majority of all crystals belong to the rhombohedral space group *R*32 (Fig. 4 ▸). The hexagonal space group was also found, with two different unit cells, referred to as *P*6_3_-large and *P*6_3_-small (Figs. 5 ▸ and 6 ▸), as was the orthorhombic space group *P*2_1_2_1_2 (Fig. 7 ▸). The latter was only observed once among the data sets collected. The crystallization conditions for each of the data sets are given in Supplementary Tables S2 and S3. Interestingly, the addition of small drug-like molecules such as cholic acid derivatives, an anaesthetic alkaloids mixture or amino acids (Gorrec, 2009 ▸, 2015 ▸; Blundell, 2017 ▸) led to the formation of new crystal packing and novel high-resolution crystal structures of C-PC, as shown and explained in Figs. 5 ▸, 6 ▸ and 7 ▸.

The crystal contacts in *P*6_3_-small are solely mediated between the α-subunits along the *ab* unit-cell plane and are arranged across the α- and β-subunits along the *c* axis, as shown in Fig. 5 ▸(*a*). Interestingly, the packing along the latter axis is also supported by a ligand, tetracaine, which was picked up from the crystallization conditions. It binds mostly via water-mediated hydrogen bonds in the cleft between the α- and β-units of the two molecular crystal planes. This particular molecule allows the formation of this specific packing as it occupies the binding site, enabling, for instance, the assembly of the dodecameric, doughnut-shaped structure that is observed in the rhombohedral and orthorhombic space groups (Fig. 5 ▸ *a*). This packing allows the generation of a solvent channel spanning the protein crystal. A loop region (Fig. 5 ▸ *b*) responsible for binding one of the covalent cofactors and another moderately rigid structure contributes to the formation of this approximately 20 Å wide pore (Figs. 5 ▸ *c* and 5 ▸ *d*).

**Figure 6:**
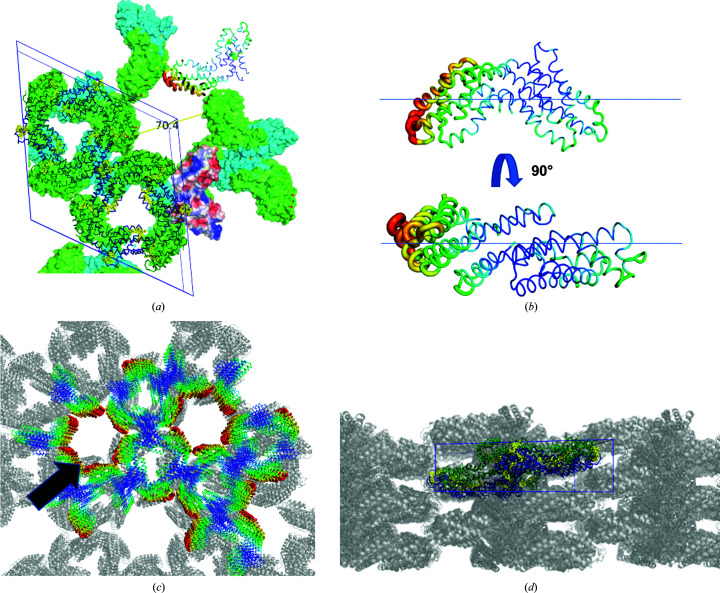
C-PC structures crystallized in space group *P*6_3_ with large unit-cell dimensions (data set 48, Supplementary Table S2). (*a*) The unit-cell content is indicated by the ribbon model facing the *ab* plane. The opening of the solvent channel was calculated and is indicated in Å. (*b*) The temperature factors are mapped onto an individual heterodimeric C-­PC protein molecule. (*c*) Flexible parts flank the open solvent channel and the black arrow indicates the solvent channel with about 70 Å diameter. (*d*) Protein packing along the *ac* axis. The individual C-PC monomers are colour-coded blue and green. The covalently bound ligands are shown in yellow.

**Figure 7:**
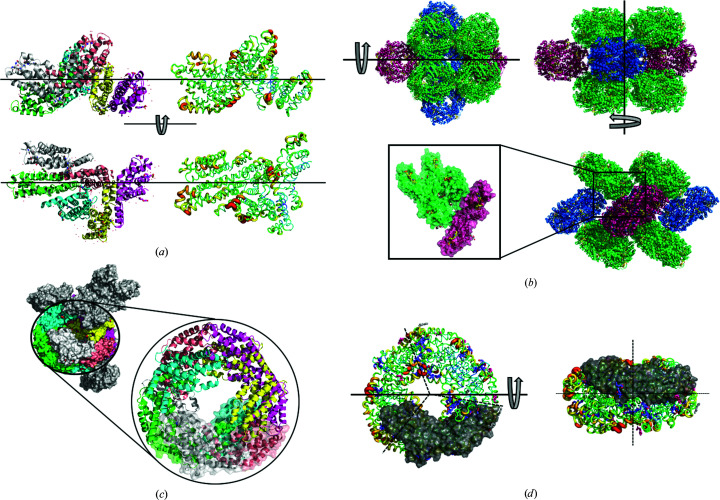
Crystal packing in the orthorhombic space group (data set 56, Supplementary Table S3). (*a*) The content of the asymmetric unit is depicted with each chain coloured individually (left) and coloured according to the determined temperature factors (right). (*b*) Six heterodimeric molecules assemble the doughnut-shaped structure, with individual colours indicating the three angles. The content of the asymmetric unit is magnified. (*c*) The content of the unit cell contains one of the dodecameric rings, and its position within the molecular arrangement is indicated. (*d*) The 120° symmetry of the ring structure is indicated by the dashed lines in the left panel. The chains are coloured according to their *B* factors, with one heterodimeric C-PC molecule shown in surface representation.

In contrast to the *P*6_3_-small unit cell, the arrangement of the molecules is slightly altered within the *P*6_3_-large unit cell. The tetrameric ring structure remains the same as a building block, but the packing is different (Figs. 5 ▸ *c* and 6 ▸ *a*). The crystal contacts are mediated by both the α- and β-subunits in each crystal lattice direction (Fig. 6 ▸ *d*). The linearly stacked rings form a tube-like structure (Figs. 6 ▸ *c* and 6 ▸ *d*). The open, accessible solvent channels have a diameter of about 70 Å and permit the diffusion of average-sized macromolecules such as peptides throughout the crystal (Erickson, 2009 ▸; Lukatsky & Shakhnovich, 2008 ▸), as depicted in Figs. 6 ▸(*a*) and 6 ▸(*c*). In the orthorhombic space group *P*2_1_2_1_2 (Fig. 7 ▸) the α-subunits solely mediate crystal contacts within the *ab* plane, and the β-subunit connects the doughnut-shaped rings in the *c* direction.

As discussed above, the addition of small molecules to the crystallization experiments creates different symmetries. The addition of an anaesthetic alkaloids mixture reshaped the pattern of preferred crystal contacts and thereby altered the crystal packing. As a result, two distinct unit cells in space group *P*6_3_ can be recognized, with the additive molecule clearly visible in the electron density of the *P*6_3_-small structure.

**Figure 8:**
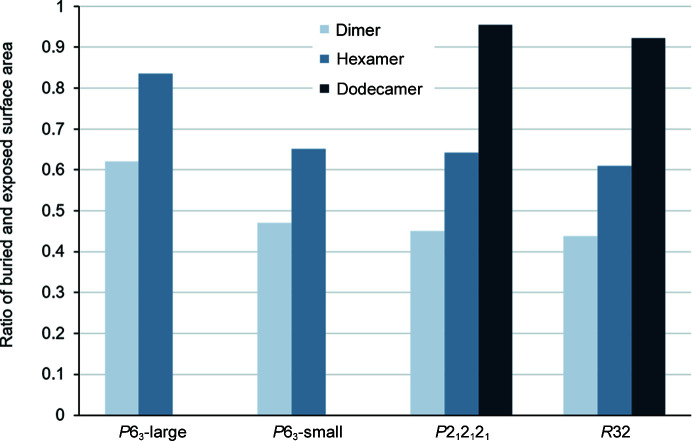
The heterodimeric building blocks assemble into higher oligomeric structures. The ratios of buried and surface-exposed areas have been calculated and are plotted for all space groups and crystal packings (Krissinel & Henrick, 2007 ▸). Values for the dimer correspond to the assembled heterodimeric αβ chain. The hexamer comprises an assembly of three heterodimers and the dodecamer is the association of two of these six-membered rings.

Analysis of the crystal packing in both hexameric space groups reveals a ring structure. As shown in Figs. 5 ▸ and 6 ▸, this is not surprising owing to the nature of the phycobilosomes and previously reported phycocyanin structures (Supplementary Table S1). In detail, the crystal interfaces between the staggered rings cover 5000 and 7180 Å^2^, with a buried-to-exposed surface ratio of 0.3 (Fig. 5 ▸ and Supplementary Fig. S8). These interfaces are specific and contribute about 50 kcal mol^−1^ per interaction and might be one driving force for this type of crystal packing. This denser packing is also reflected in the increased buried surface area as compared using the regular hexagonal building block (Supplementary Fig. S8, Fig. 8 ▸).

In contrast, the structures determined in the rhombohedral and orthorhombic space groups compose a stable dodecameric molecule. Two hexameric rings associate turned towards each other into a doughnut-shaped superstructure. The interaction surface area covers more than 61 000 Å^2^, with a ratio of surface-exposed versus buried area of almost 1 (Fig. 6 ▸ and Supplementary Fig. S8). The free energy for the assembly is calculated to be approximately 500 kcal mol^−1^ and indicates another driving force for this large stable tertiary assembly (Supplementary Fig. S7). This superstructure has not been seen in the crystal packing of any C-PC and is very likely to be connected to the natural assembly of the phycobilosome rods, although this assumption needs further in-depth investigation using other methods.

The conventionally quantified ratio of buried and solvent-accessible exposed surface area (ASA; Lee & Richards, 1971 ▸) showed a significant difference amongst the C-PC protein structures (Fig. 8 ▸). The ASA ratio in these assemblies increases, which is also a driving force in addition to the large gain in solvation free-energy gain upon the arrangement of these larger molecular structures (Fig. 8 ▸ and Supplementary Fig. S8). The specific packing of the C-PC molecules in each case might be correlated with the natural activity of C-PC as a light-harvesting antenna. Similarly, other proteins, for example chaperones or crystallines, alter their oligomeric state depending on their role in activity and their physiological environment (Jaenicke, 1996 ▸; Fu *et al.*, 2003 ▸; Libonati & Gotte, 2004 ▸).

In order to examine any structural changes in the area of the phycocyanobilin, we compared the region of the blue chromophore in all of the structures (Supplementary Table S4). In the small hexagonal space group (*P*6_3_-small), the molecular assembly of the two chains is kinked by about 6° from the chains of the structural models and the cofactor position differs by 2.8 Å from that in the other three structure (labelled 2 in Supplementary Figs. S9*b* and S9*c*). Despite this minor difference, the overall position of phycocyanobilin and the orientation of the intrinsic ligands to the respective hosts is similar in all models.

Summarizing, C-PC crystallizes in a vast number of significantly different conditions. The addition of small molecules, termed additives, gives rise to the variety of observed crystal packings. Despite the different symmetry, no notable modulation of the protein structures could be detected. This is in agreement with the observations reported for other proteins, in which changes in their oligomerization state are correlated with their activity (Jiang *et al.*, 2008 ▸).

In conclusion, the mechanisms of protein crystal packing are complex and unclear, and the effects of additives are generally not well understood (Luo *et al.*, 2018 ▸; Carugo *et al.*, 2017 ▸). In this study, we discuss the unique crystallization behaviour of C-phycocyanin, which includes effortless high-quality crystal formation with the majority of available crystallization precipitants. The effect of additives and the variation of crystal packing offers a simple new system for future in-depth investigations of protein crystallization mechanisms.

## 4. Applications and outlook   

C-phycocyanin easily produces well diffracting crystals with many morphologies, sizes and symmetries.

The molecular packing within the large *P*6_3_ unit cell, with large and open solvent channels extending over the crystalline material, could become a scaffold to accompany foreign protein molecules and facilitate the accommodation of passenger proteins in pores (Fig. 7 ▸). Consequently, crystals in this space group may become an additional tool for studying the existing scaffolds of highly porous protein crystals (Stura *et al.*, 2002 ▸; Kowalski *et al.*, 2019 ▸).

Protein crystals are routinely used, for example in technical beam alignment, detector calibration, the delivery and injection testing of crystal suspensions, investigation of the crystallization process, phasing techniques, analysing protein dynamics on a short time scale, for educational purposes and more (Haas, 2020 ▸; Yip & Ward, 1996 ▸; Norrman *et al.*, 2006 ▸; Olieric *et al.*, 2007 ▸). Most of the proteins used for methods development in protein crystallography research are available for purchase in large amounts produced from animals, unless produced recombinantly (Kim *et al.*, 2019 ▸). C-PC is produced by cyanobacteria, which grow more rapidly and are less nutritionally demanding (Yu *et al.*, 2015 ▸), and is purified using a one-column purification protocol. As one of many valuable biomolecules produced with a minimal amount of waste, it can provide crystallographers with an excellent multipurpose sample with possibilities to modify the molecular packing of crystals.

Finally, we suggest that C-PC may have applications in intermolecular cross-linking, a variety of assays and also in passenger ‘guest’ molecule imaging (Snapp, 2005 ▸). The rigid compact α-helical folding and crystal lattice may stabilize otherwise unstable proteins or support the accommodation of small molecules (Vyncke *et al.*, 2019 ▸; Ernst *et al.*, 2019 ▸). The advantageous naturally bright colour and intrinsic fluorescence make the unambiguous identification of C-PC protein crystals very convenient, particularly in experiments utilizing and scoring microcrystals (Meents *et al.*, 2017 ▸).

## 5. Related literature   

The following references are cited in the supporting information for this article: Lieske *et al.* (2019 ▸).

## Supplementary Material

PDB reference: C-phycocyanin, space group *P*6_3_, 1.45 Å resolution, 6yqg

PDB reference: 1.8 Å resolution, 6yq8

PDB reference: space group *R*32, 1.29 Å resolution, 6ypq

PDB reference: space group *P*2_1_2_1_2, 2.1 Å resolution, 6yyj

Supplementary Figures and Tables. DOI: 10.1107/S2059798320016071/nj5298sup1.pdf

## Figures and Tables

**Table 1:** Data collection and processing Values in parentheses are for the outer shell.

Structure	C-PC, *P*6_3_-large	C-PC, *P*6_3_-small	C-PC, *R*32	C-PC, *P*2_1_2_1_2
Diffraction source	P11, PETRA III, DESY	P11, PETRA III, DESY	P11, PETRA III, DESY	P11, PETRA III, DESY
Wavelength (Å)	1.0332	1.0332	1.0332	1.0332
Temperature (K)	100	100	100	100
Detector	PILATUS3 S 6M	PILATUS3 S 6M	PILATUS3 S 6M	PILATUS3 S 6M
Crystal-to-detector distance (mm)	300	160	225	160
Rotation range per image (°)	0.1	0.1	0.1	0.2
Total rotation range (°)	200	200	180	100
Exposure time per image (s)	0.05	0.05	0.05	0.05
Space group	*P*6_3_	*P*6_3_	*R*32	*P*2_1_2_1_2
*a*, *b*, *c* (Å)	153.51, 153.51, 39.36	108.20, 108.20, 66.05	187.17, 187.17, 59.93	118.36, 98.23, 104.6
α, β, γ (°)	90, 90, 120	90, 90, 120	90, 90, 120	90, 90, 90
Mosaicity (°)	0.103	0.078	0.05	0.23
Resolution range (Å)	44.32–1.82 (1.88–1.82)	46.86–1.45 (1.50–1.45)	48.19–1.29 (1.33–1.29)	47.84–2.16 (2.23–2.16)
Total No. of reflections	526799	439602	890243	488033
No. of unique reflections	48123 (4751)	77962 (7750)	99231 (9099)	65961 (6460)
Completeness (%)	99.9 (99.8)	99.9 (99.8)	99.0 (91.3)	99.4 (96.8)
Multiplicity	11.13	5.73	8.97	7.41
〈*I*/σ(*I*)〉	22.2 (0.82)	9.95 (0.53)	31.44 (4.44)	7.41 (0.69)
*R*_r.i.m._ (%)	6.1 (267)	9.6 (98.8)	3.7 (39.5)	26.1 (292)
Overall *B* factor from Wilson plot (Å^2^)	41.33	23.60	14.33	38.62

**Table 2:** Structure solution and refinement Values in parentheses are for the outer shell.

Structure name	C-PC, *P*6_3_-large	C-PC, *P*6_3_-small	C-PC, *R*32	C-PC, *P*2_1_2_1_2
Resolution range (Å)	44.31–1.82 (1.88–1.82)	46.85–1.45 (1.50–1.45)	46.79–1.29 (1.33–1.29)	47.84–2.16 (2.23–2.16)
Completeness (%)	99.85 (98.6)	99.7 (99.0)	99.0 (91.3)	99.5 (95.2)
No. of reflections, working set	48121 (4751)	77950 (7749)	99198 (9095)	65951 (6449)
No. of reflections, test set	2100 (208)	1067 (106)	1691 (155)	2098 (205)
Final *R*_cryst_	0.179 (0.35)	0.178 (0.483)	0.153 (0.244)	0.204 (0.328)
Final *R*_free_	0.201 (0.40)	0.208 (0.475)	0.170 (0.257)	0.258 (0.393)
No. of non-H atoms				
Protein	2528	2531	2613	7508
Ligand	209	148	129	485
Water	223	402	334	165
Total	2960	3081	3160	8158
R.m.s. deviations				
Bonds (Å)	0.013	0.006	0.007	0.013
Angles (°)	1.07	0.98	0.89	1.5
Average *B* factors (Å^2^)				
Overall	59.0	32.84	23.55	44.9
Protein	57.0	30.95	22.14	44.7
Ligand	84.7	35.91	21.98	50.0
Water	58.3	43.54	32.83	42.4
Ramachandran plot				
Most favoured (%)	98.2	98.17	98.47	97.25
Allowed (%)	1.5	1.83	1.53	2.75

**Table 3:** The number of conditions in which crystals appeared after 48 h SONICC and transmitted light microscope images of the 96-well plates are shown in Supplementary Figs. S3 and S4.

	No. of hits per 96 conditions	
Crystallization screen	C-PC at pH 6.5	C-PC at pH 8.0
JCSG-*plus*	82/96	76/96
Morpheus	90/96	†
Morpheus 3	95/96	63/96
Pi-Minimal	88/96	†
PACT	96/96	†
SG11	94/96	†
PGA	96/96	90/96
Midas	83/96	62/96

† Not determined.

**Table 4:** Selected examples of the diversity of crystallization conditions and indexing parameters for data collected from crystals larger than 70 µm The space group is *R*32 and the unit-cell parameters are *a* = *b* = 187, *c* = 60 Å, α = β = 90, γ = 120°. The complete results are given in Supplementary Tables S2 and S3. The protein buffer is 20 m*M* Tris pH 8.0, 100 m*M* NaCl.

Crystallization solution	pH	Maximum resolution (Å)
1.5% vitamins mix, 0.1 *M* imidazole, MES, 10% MPD, 10% PEG 1000, 10% PEG 3350	6.5	1.05
2.0 *M* ammonium sulfate, 0.1 *M* sodium acetate	4.6	1.10
0.1 *M* Bicine, 10%(*w*/*v*) PEG 6000	9	1.10
0.1 *M* ammonium sulfate, 0.3 *M* sodium formate, 0.1 *M* Tris, 3%(*w*/*v*) γ-PGA (Na^+^ form, LM), 10%(*w*/*v*) PEG 2000 MME	7.8	1.12
0.3 *M* potassium bromide, 0.1 *M* sodium acetate, 8%(*w*/*v*) γ-PGA (Na^+^ form, LM)	5	1.16
40% PEG 300, 100 m*M* phosphate–citrate	4.5	1.18
0.2 *M* magnesium chloride hexahydrate, 0.1 *M* MES, 14%(*v*/*v*) pentaerythritol propoxylate (17/8 PO/OH)	5.5	1.19
0.2 *M* potassium citrate tribasic monohydrate, 15%(*w*/*v*) Sokalan CP 42	—	1.22
0.1 *M* MES, 30%(*w*/*v*) poly(acrylic acid sodium salt) 5100, 10% ethanol	6	1.25
0.2 *M* zinc acetate dihydrate, 0.1 *M* sodium acetate, 10%(*w*/*v*) PEG 3000	4.5	1.26
0.1 *M* Tris, 5%(*w*/*v*) γ-PGA (Na^+^ form, LM), 20%(*w*/*v*) PEG 3350	7.8	1.26
0.2 *M* sodium chloride, 0.1 *M* Bicine, 20%(*w*/*v*) poly(acrylic acid sodium salt) 2100	9	1.37
0.1 *M* MES, 12% polyvinylpyrrolidone	5.5	1.54

**Table 5:** Summary of the results from the X-ray diffraction data collected from single crystals For C-PC at pH 6.5, 58 data sets were analyzed and for C_PC at pH 8.0, there were 60 data sets. The complete results are given in Supplementary Tables S2 and S3.

	No. of data sets	
Data collection/indexing	C-PC at pH 6.5	C-PC at pH 8.0
Resolution < 1.2 Å	17/58	24/60
Resolution < 1.35 Å	28/58	36/60
Resolution < 2 Å	53/58	53/60
Space group *R*32	53/58	47/60
Space group *P*6_3_	3/58	11/60
Other space groups	3/58	2/60
