# Supplementary material for: C-phycocyanin as a highly attractive model system in protein crystallography: unique crystallization properties and packing-diversity screening
Source: Acta Crystallogr D Struct Biol. 2021 Jan 26;77(Pt 2):224–36. doi: 10.1107/S2059798320016071 (PMC7869899; doi:10.1107/S2059798320016071)
Supplement: Supplementary file 1 [file d-77-00224-sup1.pdf]

STRUCTURAL BIOLOGY

Volume 77 (2021)

Supporting information for article:

C-phycocyanin as a highly attractive model system in protein crystallography, unique crystallization properties and packing-diversity screening

Iosifina Sarrou, Christian G. Feiler, Sven Falke, Nolan Peard, Oleksandr Yefanov and Henry Chapman

Table S1 Crystal structures of C-phycocyanin from cyanobacteria.

	PDB ID	Source	Max. resolution	Symmetry	Precipitant	Unit cell parameters
1	4ZIZ	*T.elongatus*	1.75Å	H32	PEG 3350, HEPES pH 7.0	186, 186, 60<br>90, 90, 120
2	4Z8K	*T. elongatus*	2.5Å	$P6_3$	Ammonium sulfate, MES pH 6.1	153, 153, 39<br>90, 90, 120
3	4H0M	*T.elongatus PCC7942*	2.2Å	P1 2_1 1	PEG 4000<br>Tris pH 8.0	106, 113, 184<br>90, 90, 90
4	1JBO	*T.elongatus*	1.45Å	H32	Ammonium sulfate, MES pH 6.1	188, 188, 60<br>90, 90, 120
5	4N6S	*T. vulcanus*	2.4Å	H32	1.4 M phosphate buffer	188, 188, 60<br>90, 90, 120
6	4GXE<br>4GY3	*T. vulcanus*	3.0Å<br>2.5 Å	$P6_3$	Ammonium sulfate, Tris pH 8.0	153, 153, 39<br>90, 90, 120
7	3O18	*T.vulcanus*	1.35Å	H32	Ammonium sulfate, sucrose, Tris pH 8.0	186, 186, 60<br>90, 90, 120
8	3O2C	*T.vulcanus*	1.5Å	H32	1.2 M phosphate buffer	187, 187, 60<br>90, 90, 120
9	1ON7	*T.vulcanus*	2.7Å	$P6_3$	PEG4000, bis Tris pH 7.0	153, 153, 39<br>90, 90, 120
10	5TOU	*Pseudanabaena sp. lw0831*	2.04 Å	$P2_1$ 2_1 2_1	PEG 4000,<br>sodium acetate pH 5.0	67, 175, 194<br>90, 90, 90
11	4L1E	*Leptolyngbya sp. N62DM*	2.61Å	C121	PEG 2000,<br>Tris pH 9.0	183, 107, 111<br>90, 98, 90
12	4F0T	*Synechocystis sp. PCC 6803*	2.61Å	$P6_3$	PEG 4000, $MgSO_4$,<br>Tris pH 8.0	153, 153, 40<br>90, 90, 120
13	1HA7<br>1GH0	*Arthrospira platensis*	2.2Å	P1 2_1 1	PEG 6000, 10% EtOH pH 6.8	107, 115, 183<br>90, 90, 90
14	1CPC	*Microchaete diplosiphon*	1.66Å	R3	10 to 12.5% PEG,<br>0.1 M phosphate<br>pH 5.0	180, 180, 61<br>90, 90, 120

C-Phycocyanin microcrystals appear to have an advantage as a model protein for easy visualization in serial crystallography experiments. As shown in figure S1A and B, the crystal density on a fix target chip can be easily adjust. The microcrystals shown in (A, B) were grown with the batch method and appear in few hours.

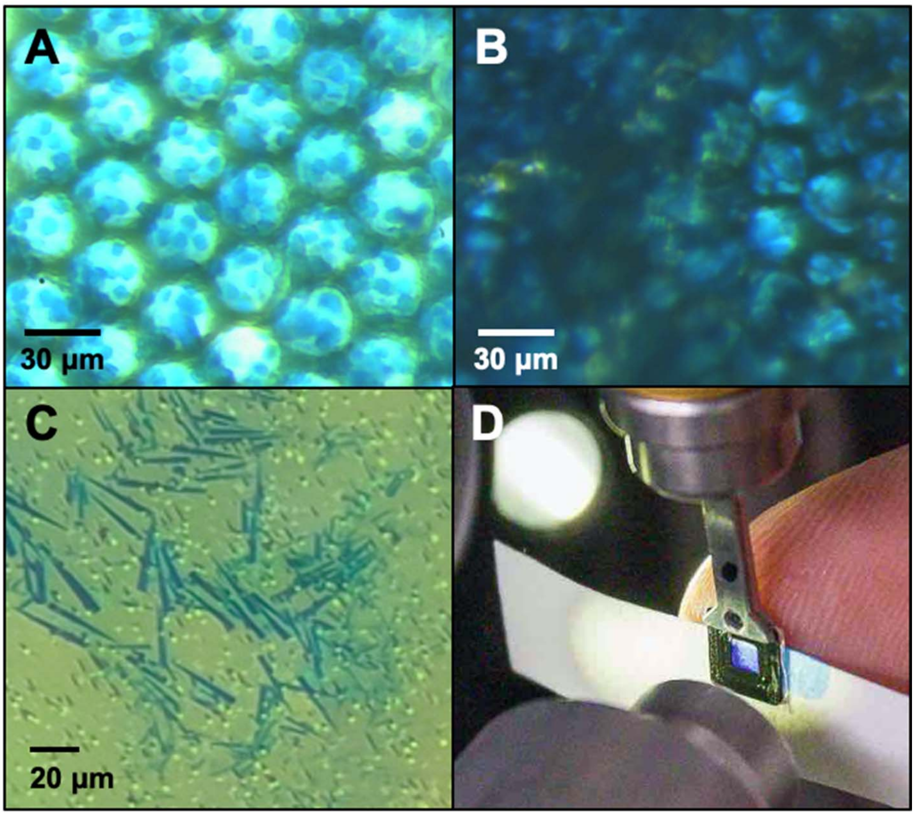

Figure S1 (a and b) Microcrystalline material loaded on a silicon chip (Lieske et al., 2019) and used in a serial crystallography setup. The natural blue color makes the crystal density on the chip easily adjusted. Microcrystals diffracted up to 2.3 Å resolution (Meents et al., 2017). The diameter of a single hole is 30 µm and crystals do not exceed 10 µm diameter. (c) Self-assembled crystalline material of larger particle size (formed in solution in acetate buffer pH4) loaded onto an XtalTool-HT (Feiler et al., 2019) and data were collected with the serial crystallography approach. (d) Application of C-PC microcrystals for serial crystallography on silicon chip directly at the beam. The mother liquid and nanocrystal are blotted before the data collection.

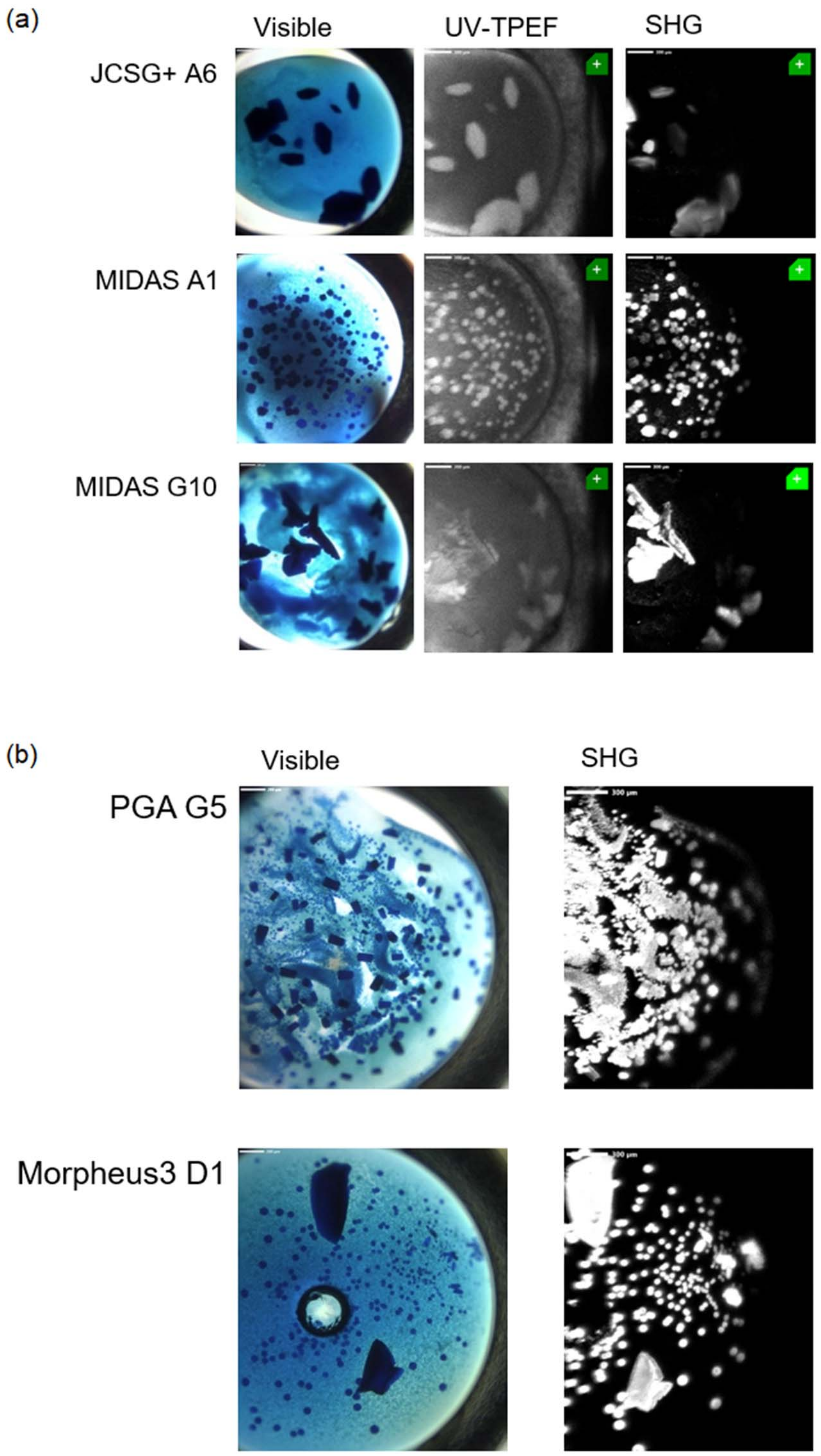

Figure S2 (a) Images of randomly picked crystals in UV-TEF and SONICC. The images show that the SHG signal is not enhanced by the chromophore present in the protein therefore there is not a false positive signal during imaging. (b) Examples of droplets were two crystal sizes are appearing, therefore in these cases, we included both sizes in the statistics shown in Figure 2(a).

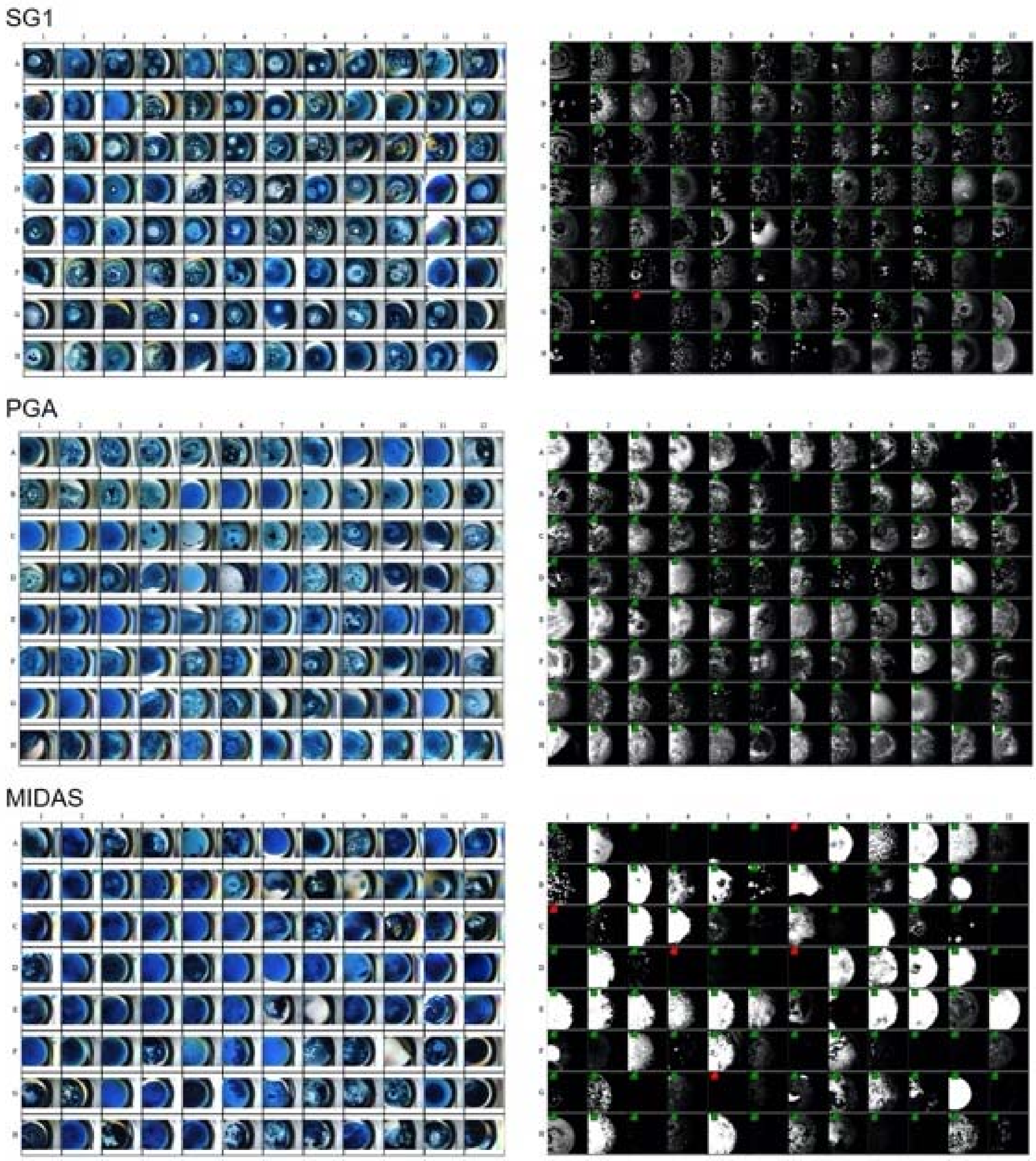

Figure S3 Images of another three 96 well plates in visible (left) and the SHG imaging mode (right) in continuation of figure 3. The details on the plates are described in table 3 (protein buffered at pH 6.5) and the text. To examine the possibility of a false positive SHG signal due to the presence of the chromophore in C-PC or a higher symmetry space group, UV-TEF imaging was additionally utilized, which is based on intrinsic tryptophan fluorescence (see examples in figure S2).

Screen JCSG

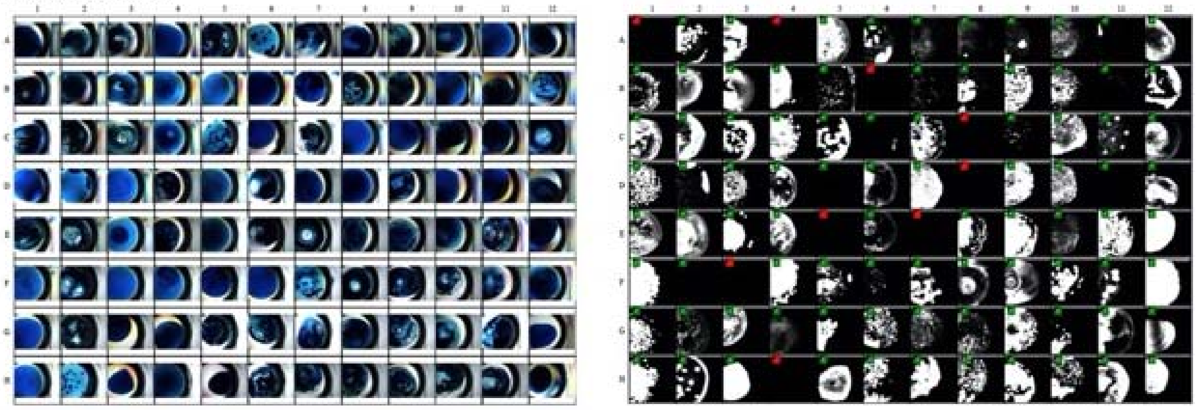

Screen Morpheus 3

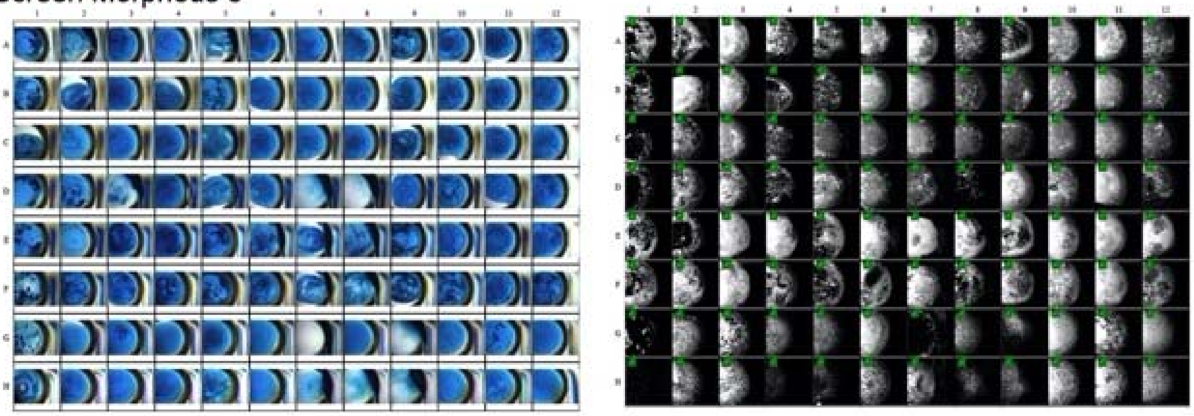

Screen PACT

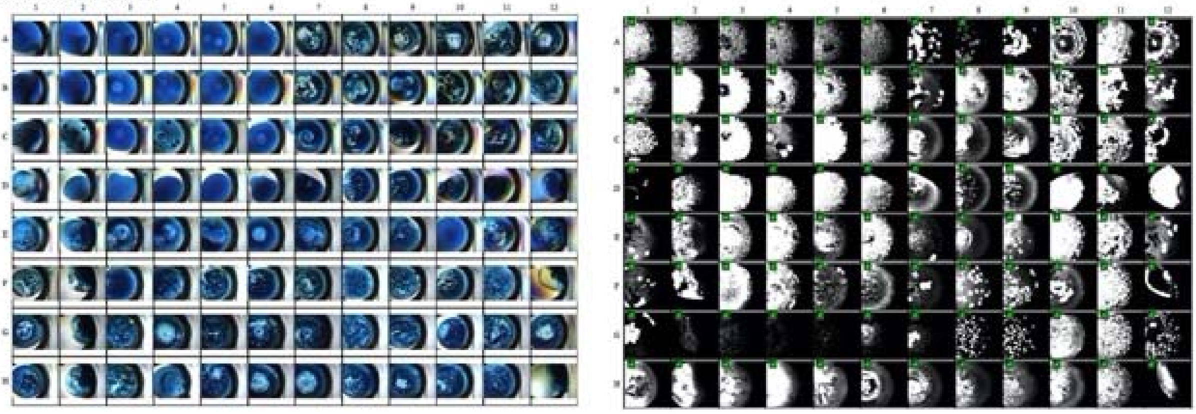

Figure S4 Images of the 96 well plates in visible light (left) and the SHG imaging mode (right). The second harmonic generation imaging, as shown on the right side, is positive, i.e., light is emitted when chiral crystals are present in the droplet. To examine the possibility of a false positive due to the presence of the chromophore in C-PC or a higher symmetry space group UV-TPEF imaging was additionally utilized, which is based on intrinsic tryptophan fluorescence (see examples in figure S2).

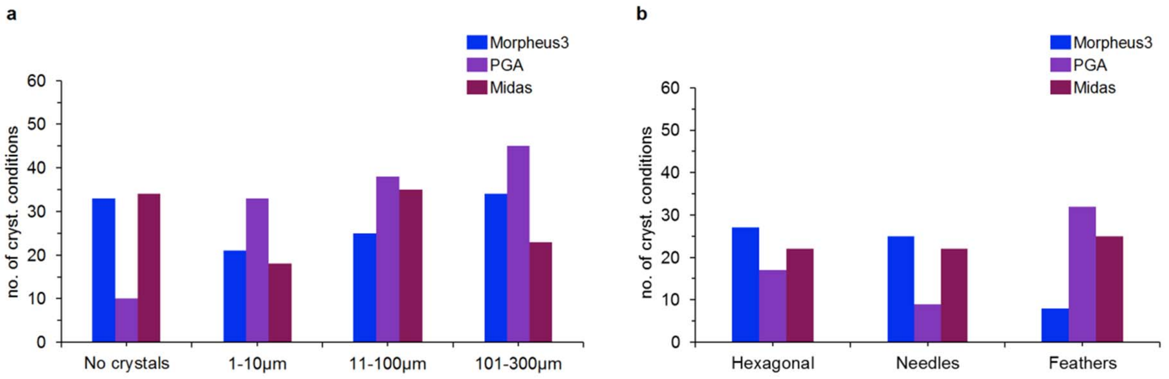

Figure S5 The size distribution of crystals as they appear under different conditions. Naturally, within one crystallization drop, crystal sizes may vary, the results are determined by the observations of the majority of crystals. In some cases, when the crystals have two distinct size regimes, both are included, see examples in figure S2. (b) Three categories of crystal morphology in the C-PC crystallization experiments utilizing three different screens as shown in table 1, with C-PC in Tris buffer at pH 8.0. Please note that the morphologies are reported for crystals bigger than 10 µm.

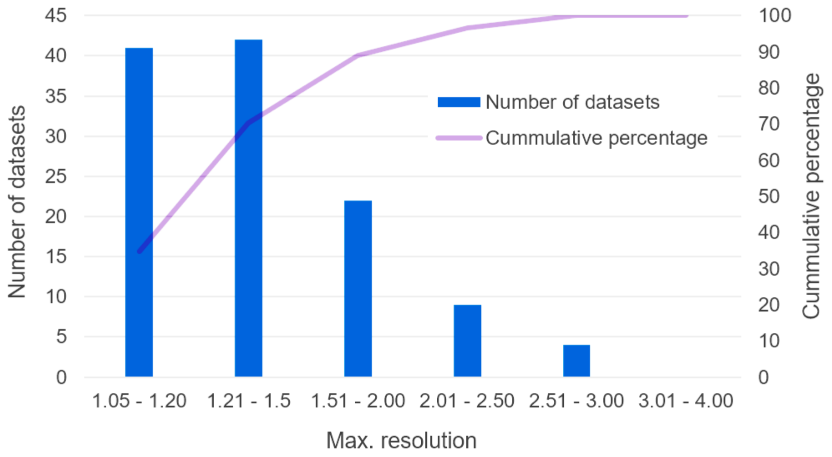

Figure S6 A total number of 118 datasets were analysed. The data were categorized into six different resolution classes. The cumulative percentage of individual categories is reflected. The number of individual datasets populating the different maximum resolution bins in Å is provided.

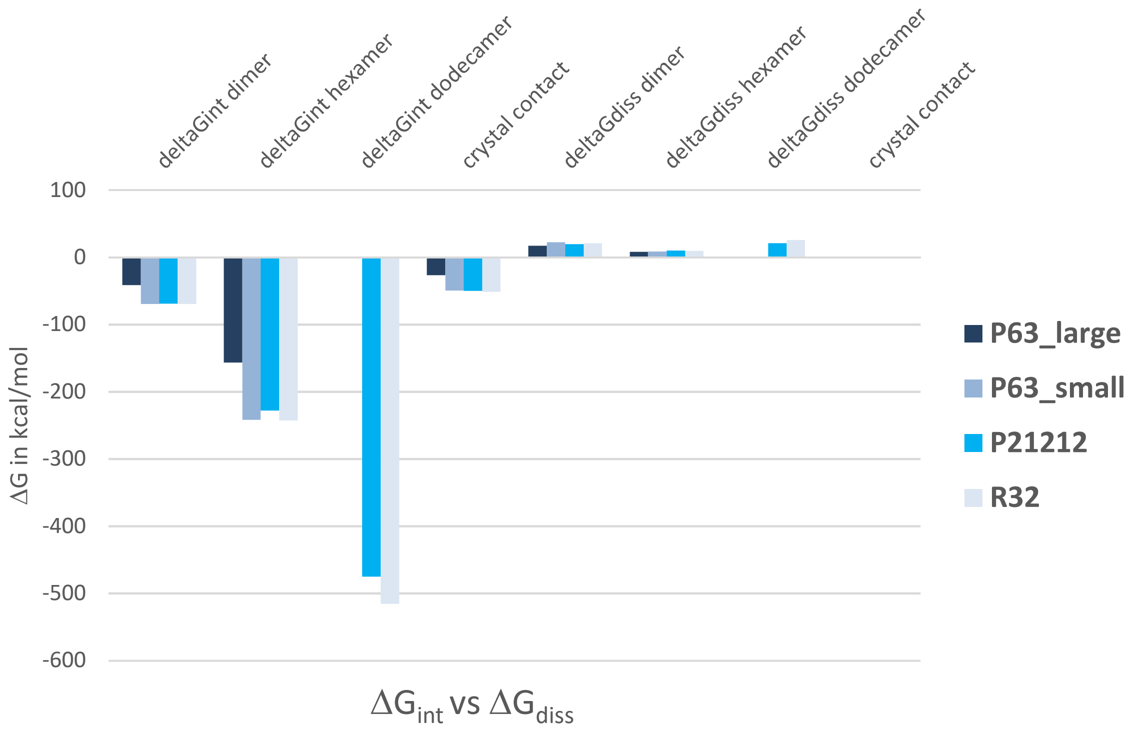

Figure S7 Individual ΔG_{int}, and ΔG_{diss}, calculated in kcal/mol, indicate the solvation free energy gain upon formation of the individual assembly of the C-PC molecules and the individual assembly dissociation, respectively, ΔG_{int} is the difference in total solvation energies of isolated and assembled molecules without taking the effect of satisfied hydrogen bonds and salt bridges across the interfaces into account. The positive values of ΔG_{diss} indicate that external driving forces are needed to be applied to dissociate the given assembly. Values of $\Delta G_{diss}>0$ indicate thermodynamically stable complexes.

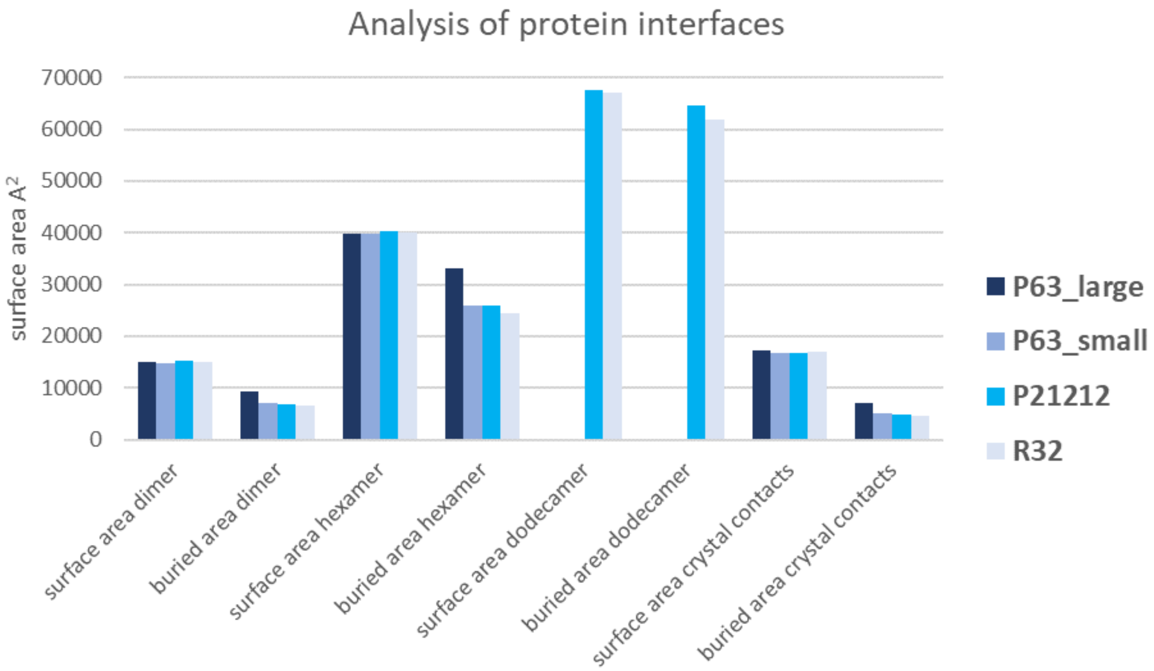

Figure S8 Calculated Surface areas are plotted for each observed space group and indicate the solvent-accessible protein surface area of individual assemblies. The buried area depicts the solvent-accessible surface area of monomeric units buried upon the assembly of hexameric or dodecameric structures. All surface areas are calculated in square Å.

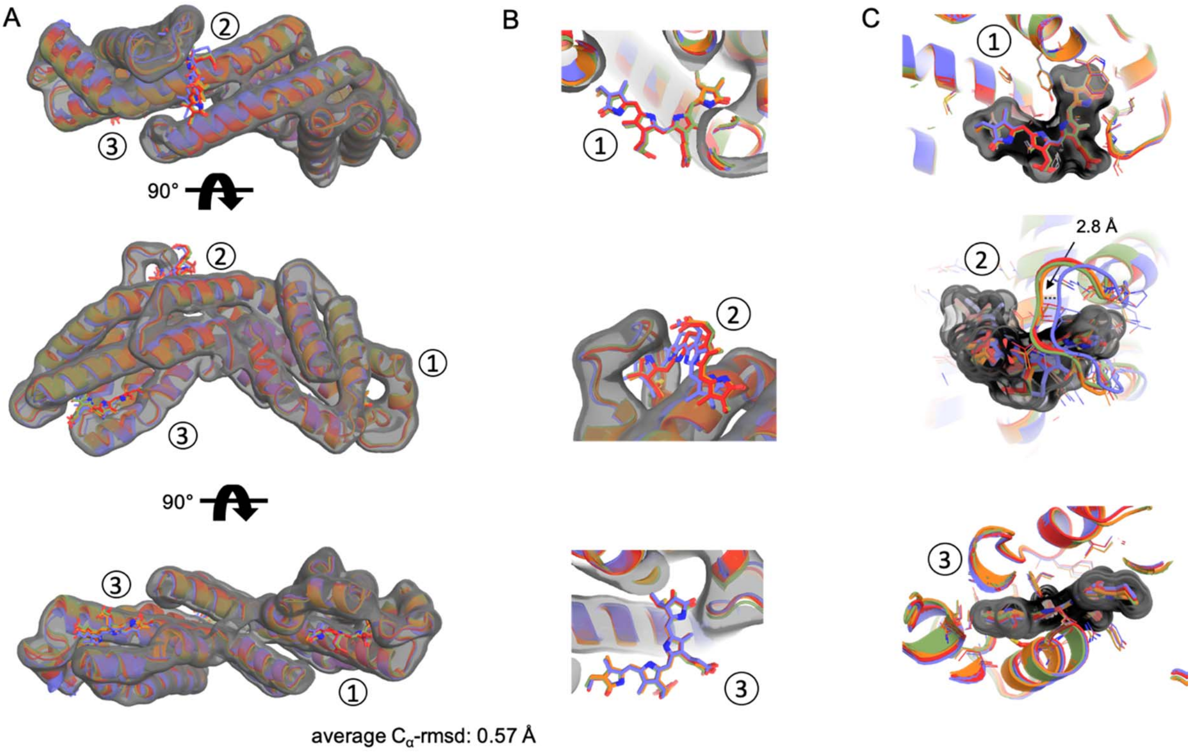

Figure S9 (a) Superposition of all structural models. The average C_α rmsd calculated to 0.57 Å is slightly higher than the structural coordinate error. All models are individually coloured and the positions of the phycocyanobilin cofactor are numbered through-out all panels. (b) A magnified stick representation of the cofactor and its binding region in cartoon representation is shown for each of the three ligand molecules. (c) The ligand binding pockets were calculated and are shown for each structure is superposition with the occupying ligand in stick representation. The dotted line in the middle panel indicates a 2.8 Å difference in the ligand binding position.

Table S2 Indexing parameters on data collected from crystals larger than 70 µm.

The protein was in 20 mM TRIS pH 8.0, 100 mM NaCl. The crystallization screens used is mentioned on the first column and the number from A1 to H12 corresponds to the drops on a 96 well plate MRC2. The crystallization experiments were set up manually with the mixing of 1 µl protein and 1 µl precipitant. All the plates were stored at 20 °C unless is mentioned otherwise. The crystals were randomly picked and froze in 25% PEG400 when cryoprotectant was necessary.

No	Screen	SPG no	a [Å]	b [Å]	c [Å]	α [deg]	β [deg]	γ [deg]	Maxres [Å]	pH	Crystallization solution
**1**	Morpheus3 B4	155	186.8	186.8	59.96	90	90	120	1.05	6.5	1.5% Vitamins Mix. 0.1 M Buffer System1. 50% Precipitant Mix 4
**2**	Morpheus3 H1	155	187.31	187.31	59.92	90	90	120	1.05	6.5	0.8% Anaesthetic alkaloids mix. 0.1 M Buffer System1 .50% Precipitant mix1
**3**	Morpheus3 D1	155	187.23	187.23	59.7	90	90	120	1.07	6.5	0.35% Phytochemicals 1 mix; 0.1 M Buffer System 1; 50% Precipitant Mix 1
**4**	Morpheus3 E1	155	187.22	187.22	59.85	90	90	120	1.07	6.5	0.25% Phytochemicals 2 mix; 0.1 M Buffer System 1; 50% Precipitant Mix 1
**5**	Morpheus3 A1	155	186.98	186.98	59.74	90	90	120	1.08	6.5	1.6% Dipeptide Mix. 0.1 M Buffer System1. 50% Precipitant Mix 1
**6**	JCSG C11	155	187.28	187.28	59.84	90	90	120	1.10	4.6	2.0 M Ammonium sulfate; 0.1 M Sodium acetate
**7**	JCSG E10	155	187.38	187.38	60.11	90	90	120	1.10	9	0.1 M Bicine; 10% w/v PEG 6000
**8**	JCSG H2	155	187.02	187.02	59.97	90	90	120	1.10	-	1.0 M Ammonium sulfate; 0.1 M BIS-Tris; 1% w/v PEG 3350
**9**	JCSG A3	155	187.01	187.01	59.94	90	90	120	1.12	-	0.2 M Ammonium citrate dibasic; 20% w/v PEG 3350
**10**	PGA H8	155	187.64	187.64	59.87	90	90	120	1.12	7.8	0.1 M Ammonium sulfate; 0.3 M Sodium formate; 0.1M Tris; 3% w/v γ-PGA (Na+ form. LM); 10% w/v PEG 2000 MME
**11**	JCSG C4	155	186.89	186.89	59.72	90	90	120	1.13	7	0.1M HEPES; 10% w/v PEG 6000

**12**	MIDAS H11	155	187.08	187.08	59.69	90	90	120	1.13	-	0.2 M Ammonium formate; 10% w/v Polyvonylpyrrolidone; 20% w/v PEG 4000
**13**	Morpheus3 F1	155	187.19	187.19	60.12	90	90	120	1.13	6.5	0.6% Antibiotics mix; 0.1M Buffer System 1; 50% Precipitant Mix 1
**14**	Morpheus3 F4	155	186.63	186.63	60.13	90	90	120	1.14	6.5	0.6% Antibiotics Mix. 0.1M Buffer System1. 50% Precipitant Mix4
**15**	JCSG G9	155	187.27	187.27	60	90	90	120	1.15		0.1 M pottasium thiocyanine. 30% PEG2000MME
**16**	PGA A1	155	186.97	186.97	59.85	90	90	120	1.16	5	0.3 M Potassium bromide; 0.1 M Sodium acetate; 8% w/v γ-PGA (Na+ form. LM)
**17**	PGA E10	155	187.5	187.5	60	90	90	120	1.16	6.5	0.1M Ammonium sulfate; 0.3 M Sodium formate; 0.1M Sodium cacodylate; 3% w/v γ-PGA (Na+ form. LM); 20% v/v PEG 500 MME
**18**	PGA A2	155	187.36	187.36	59.73	90	90	120	1.17	5	0.2 M Magnesium chloride; 0.1 M Sodium acetate; 8% w/v γ-PGA (Na+ form. LM)
**19**	JCSG C6	155	153.8	153.8	39.61	90	90	120	1.18	4.5	40% PEG300 100mM Phosphate/citrate
**20**	JCSG A9	155	187.73	187.73	60.08	90	90	120	1.19	-	0.2 M Ammonium chloride; 20% w/v PEG 3350
**21**	JCSG H8	155	187.03	187.03	59.63	90	90	120	1.19	5.5	0.2 M Sodium chloride; 0.1 M BIS-Tris; 25% w/v PEG 3350
**22**	JCSG D2	155	186.79	186.79	59.73	90	90	120	1.19	7.5	0.2 M Magnesium chloride hexahydrate; 0.1 M Sodium HEPES; 30% v/v PEG 400
**23**	MIDAS A5	155	187.35	187.35	60.1	90	90	120	1.19	-	0.5 M Ammonium phosphatemonobasic; 12.5% w/v Poly (acryl acid sodium salt) 2100
**24**	MIDAS F8	155	186.76	186.76	59.49	90	90	120	1.19	5.5	0.2 M Magnesium chloride hexahydrate; 0.1 M MES; 14% v/v Pentaerythritol propoxylate (17/8 PO/OH)

**25**	MIDAS H7	155	187.41	187.41	60.14	90	90	120	1.22	-	0.2M Potassium citrate tribasic monohydrate; 15% w/v SOKALAN CP 42
**26**	MIDAS E11	155	187.29	187.29	60.28	90	90	120	1.23	6.5	0.1 M Lithium sulfate; 0.1 M HEPES; 25% w/v Poly (acryl acid sodium salt) 2100
**27**	MIDAS H6	155	187.6	187.6	60.37	90	90	120	1.25	6	0.1 M MES; 30% w/v Poly (acrylic acid sodium salt) 5100. 10% Ethanol
**28**	JCSG C7	155	186.62	186.62	59.76	90	90	120	1.26	4.5	0.2 M Zinc acetate dihydrate; 0.1 M Sodium acetate; 10% w/v PEG 3000
**29**	JCSG H9	155	187.61	187.61	60.05	90	90	120	1.26	5.5	0.2 M Lithium sulfate; 0.1 M BIS-Tris; 25% w/v PEG 3350
**30**	JCSG B2	155	186.79	186.79	59.71	90	90	120	1.26	-	0.2 M Sodium thiocyanate; 20% w/v PEG 3350
**31**	PGA A12	155	187.53	187.53	60.16	90	90	120	1.26	5	0.1 M Sodium acetate; 5% w/v γ-PGA (Na+ form. LM); 20% w/v PEG 2000 MME
**32**	PGA G5	155	186.81	186.81	59.54	90	90	120	1.26	7.8	0.1 M Tris; 5% w/v γ-PGA (Na+ form. LM); 20% w/v PEG 3350
**33**	PGA B3	155	186.93	186.93	59.95	90	90	120	1.29	5	0.1M Sodium acetate; 5% w/v γ-PGA (Na+ form. LM); 12% w/v PEG 8000
**34**	PGA D9	155	187.31	187.31	60.03	90	90	120	1.31	6.5	0.1 M Sodium cacodylate; 5% w/v γ-PGA (Na+ form. LM); 20% w/v PEG 3350
**35**	Morpheus3 F7	173	154.24	154.24	39.59	90	90	120	1.33	7.5	0.6% antibiotics mix; 0.1M Buffer System 2; 50%precipitant mix 3
**36**	JCSG A2	155	187.25	187.25	59.83	90	90	120	1.35	5.5	0.1 M Sodium citrate; 20% w/v PEG 3000
**37**	MIDAS F4	155	187.5	187.5	60.18	90	90	120	1.37	9	0.2 M Sodium chloride; 0.1 M BICINE; 20% w/v Poly(acrylic acid sodium salt) 2100
**38**	JCSG C5	155	187.24	187.24	60.13	90	90	120	1.39	7.5	0.8 M Sodium phosphate monobasic monohydrate; 0.1 M Sodium HEPES

**39**	JCSG E8	155	186.35	186.35	59.88	90	90	120	1.41	4.5	1.0 M Ammonium phosphate dibasic; 0.1 M Sodium acetate
**40**	JCSG G6	155	187.18	187.18	59.72	90	90	120	1.42	-	0.2 M Sodium malonate dibasic monohydrate; 20% w/v PEG 3350
**41**	JCSG A8	155	187.16	187.16	59.58	90	90	120	1.43	-	0.2 M Ammonium formate; 20% w/v PEG 3350
**42**	Morpheus3 H6	173	108.12	108.21	66.05	90	90	120	1.45	7.5	0.8% Anaesthetic alkaloids mix. 0.1 M Buffer System2 .50% Precipitant mix2
**43**	JCSG E9	155	187.42	187.42	60.69	90	90	120	1.46	6.5	1.6 M Magnesium sulfate heptahydrate; 0.1 M MES
**44**	JCSG G8	155	187.26	187.26	60.25	90	90	120	1.47		0.15 M d-l malic acid. 20% PEG3350
**45**	JCSG E4	155	187.19	187.19	60.44	90	90	120	1.49	8.5	1.26 M Ammonium sulfate; 0.1 M Tris
**46**	JCSG H1	155	187.08	187.08	59.97	90	90	120	1.54	5.5	0.3 M Magnesium formate dihydrate; 0.1M BIS-Tris
**47**	MIDAS A2	155	186.06	186.06	59.82	90	90	120	1.54	5.5	0.1 M MES. 12% polyvinylpyrrolidone
**48**	Morpheus3 C11	173	153.51	153.51	39.36	90	90	120	1.82	8.5	1% Nucleosides Mix. 0.1 M Buffer System3. 50% Precipitant Mix 3
**49**	Morph3 G6	4	151.64	39.01	157.87	90	116.9	90	1.87	7.5	1.2% Cholic Acid derivatives mix. 0.1 M Buffer System2. 50% precipitant Mix2
**50**	JCSG F10	155	184.11	184.11	58.59	90	90	120	1.89	7	1.1 M Sodium malonate dibasic monohydrate; 0.1 M HEPES; 0.5% v/v Jeffamine ED-2003
**51**	Morpheus3 F6	173	152.85	152.85	39.28	90	90	120	1.91	7.5	0.6% Antibiotics mix; 0.1 M Buffer System 2; 50% Precipitant Mix 2
**52**	Morpheus3 F10	173	153.8	153.8	39.61	90	90	120	1.94	8.5	0.6% antibiotics mix; 0.1 M Buffer System 2; 50%precipitant mix 3
**53**	Morpheus3 H7	173	153.58	153.58	39.44	90	90	120	2.03	7.5	0.8% Anesthetic alkaloids mix. 0.1M Buffer System2. 50% Precipitant mix3
**54**	Morpheus3 A8	173	152.49	152.49	39.26	90	90	120	2.04	7.5	1.6% Dipeptide Mix. 0.1 M Buffer System2. 50% Precipitant Mix 4

**55**	Morpheus3 B2	173	153.69	153.69	39.32	90	90	120	2.10	6.5	1.5% Vitamins Mix. 0.1 M Buffer System1. 50% Precipitant Mix 2
**56**	Morpheus3 B12	173	152.54	152.54	39.19	90	90	120	2.11	8.5	1.5% Vitamins Mix. 0.1 M Buffer System3. 50% Precipitant Mix 4
**57**	Morpheus3 C3	173	152.65	152.65	39.22	90	90	120	2.23	6.5	1% Nucleosides mix; 0.1 M Buffer System 1; 50% Precipitant Mix 3
**58**	Morpheus3 F2	173	154.43	154.43	39.61	90	90	120	2.24	6.5	0.6% Antibiotics Mix. 0.1M Buffer System1. 50% Precipitant Mix2
**59**	JCSG H3	1	59.82	109.93	122.04	112.98	96.29	100.12	2.66	5.5	0.1 M BIS-Tris; 25% w/v PEG 3350
**60**	Morpheus3 H2	173	154.17	154.17	39.25	90	90	120	2.68	6.5	0.8% Anesthetic alkaloids mix; 0.1 M Buffer System 1; 50% Precipitant Mix 2

Table S3 Indexing parameters on data collected from crystals larger than 70 µm.

The protein was in 20 mM MES pH 6.5 100 mM NaCl. The crystallization screens used is mentioned on the first column and the number from A1 to H12 corresponds to the drops on a 96 well plate MRC2. The crystallization experiments were set up manually with the mixing of 1 µl protein and 1 µl precipitant. All the plates were stored at 20 °C unless is mentioned otherwise. The crystals were randomly picked and froze in 25% PEG 400 when cryoprotectant was necessary.

No	Screen	SPG no	a [Å]	b [Å]	c [Å]	α [deg]	β [deg]	γ [deg]	Maxres [Å]	pH	Crystallization solution
**1**	JCSG B1	155	187.65	187.65	60.1	90	90	120	1.07	4.0	0.8 M Ammonium Sulfate. 0.1 M Citrate
**2**	Morph3 E1	155	187.22	187.22	59.85	90	90	120	1.07	6.5	0.25% Phytochemicals 2 mix. 0.1 M Buffer System1. 50% precipitant Mix1
**3**	JCSG C4	155	187.01	187.01	60.11	90	90	120	1.1	7.0	0.1 M HEPES; 10% w/v PEG 6000
**4**	JCSG F1	155	187.36	187.36	60.08	90	90	120	1.1	6.5	0.05 M CesiumChloride. 0.1 M MES. 30% Jeffamine 600
**5**	JCSG G1	155	186.91	186.91	60.03	90	90	120	1.1	7.5	0.1 M HEPES. 30% Jeffamine ED2003
**6**	MIDAS G7	155	186.95	486.95	60.14	90	90	120	1.1	6.5	0.2 M Ammonium acetate; 0.1 M MES; 30% v/v Glycerol ethoxylate
**7**	Morph3 F1	155	187.41	187.41	59.94	90	90	120	1.1	6.5	0.6% antibiotics mix; 0.1 M Buffer System 1; 50% precipitant mix1
**8**	Morph3 H1	155	187.21	187.21	59.74	90	90	120	1.1	6.5	0.8% anesthetic alkaloid mix; 0.1 M Buffer System 1; 50% precipitant mix1
**9**	MIDAS C2	155	187.07	187.07	59.98	90	90	120	1.12	6.0	0.2 M Sodium chloride; 0.1 M MES; 30% v/v Jeffamine ED-2003
**10**	Morpheus H1	155	187.17	187.17	59.93	90	90	120	1.13	6.5	0.1 M Amino acids. 0.1 M buffer system 1. 30% v/v P500MME_P20K
**11**	Morpheus H5	155	187.16	187.16	59.94	90	90	120	1.15	7.5	0.1 M Amino acids. 0.1 M buffer system 2. 30% v/v P500MME_P20K
**12**	Morph3 H5	155	187.28	187.28	59.98	90	90	120	1.15	7.5	0.8% anesthetic alcaloid mix; 0.1 M buffer system 2; 50% precipitant mix1
**13**	PGA B2	155	186.85	186.85	59.78	90	90	120	1.16	5.0	0.1 M Sodium acetate; 5% w/v γ-PGA (Na+ form. LM); 15% w/v PEG 4000

**14**	JCSG B7	155	187.11	187.11	59.89	90	90	120	1.16	4.6	0.1 M Sodium acetate; 8% w/v PEG 4000
**15**	JCSG E1	155	187.79	187.79	60.28	90	90	120	1.19	6.5	1 M Sodium Citrate tribasic dehydrate. 0.1 M MES
**16**	PACT A6	155	187.37	187.37	60.14	90	90	120	1.19	9.0	0.1 M SPG; 25% w/v PEG 1500
**17**	PACT H2	155	186.81	186.81	59.88	90	90	120	1.19	8.5	0.2 M Sodium bromide; 0.1M Bis-Tris propane; 20% w/v PEG 3350
**18**	Morpheus B1	155	186.9	186.9	60.08	90	90	120	1.21	6.5	0.09 M Halogens. 0.1 M buffer system1. 30%v/v P500MME_P20K
**19**	Morph3 F5	155	187.28	187.28	60.24	90	90	120	1.22	7.5	0.6% antibiotics mix; 0.1M buffer system 2; 50% precipitant mix1
**20**	JCSG F9	155	187.24	187.24	60.21	90	90	120	1.23	7.0	2.4 M Sodium Malonate dibasic monohydrate
**21**	Morph3 F4	155	186.87	186.87	60.03	90	90	120	1.23	6.5	0.6% antibiotics mix; 0.1 M Buffer System 1; 50% precipitant mix4
**22**	Morpheus C1	155	187.07	187.07	60	90	90	120	1.24	6.5	0.09 NPS. 0.1 M Buffer System1. 30% v/v P500MME_P20K
**23**	Morpheus B5	155	187.53	187.53	60.14	90	90	120	1.25	7.5	0.09 M Halogens. 0.1 M buffer System2. 30% v/v P500MME_P20K
**24**	PGA G5	155	186.88	186.88	59.96	90	90	120	1.25	7.8	0.1 M Tris; 5% w/v γ-PGA (Na+ form. LM); 20% w/v PEG 3350
**25**	PACT C11	155	186.88	186.88	60.03	90	90	120	1.25	7.0	0.2 M Calcium chloride dihydrate; 0.1 M HEPES; 20% w/v PEG 6000
**26**	Morpheus A11	155	187.07	187.07	60.19	90	90	120	1.31	8.5	0.06 M Divalents. 0.1M Buffer system 3. 30% v/v GOL_P4K
**27**	MIDAS A1	155	186.8	59.88	59.95	90	90	120	1.31	6.0	0.1 M HEPES; 50% v/v Polypropylene glycol 400
**28**	PACT C3	155	187.08	187.08	59.83	90	90	120	1.34	6.0	0.1 M PCTP; 25% w/v PEG 1500
**29**	PGA A1	155	187.06	187.06	60.2	90	90	120	1.37	5.0	0.3 M Potassium bromide; 0.1 M Sodium acetate; 8% w/vγ-PGA (Na+ form. LM)
**30**	MIDAS D3	155	186.93	186.93	59.92	90	90	120	1.37	-	45% v/v Polypropylene glycol 400. 10% Ethanol
**31**	JCSG A8	155	187.15	187.15	59.9	90	90	120	1.39		0.2 M Ammonium formate; 20% w/v PEG 3350

**32**	JCSG F5	155	188.3	188.3	60.45	90	90	120	1.4	8.5	0.2 M Magnesium Chloride hexahydrate. 0.1M Tris. 50% Ethylene glycol
**33**	MIDAS A9	155	187.01	187.01	60.04	90	90	120	1.4	6.0	0.1 M MES; 25% v/v Pentaerythritol propoxylate (5/4 PO/OH)
**34**	PGA D12	155	186.47	186.47	59.72	90	90	120	1.42	6.5	0.1 M Sodium cacodylate; 5% w/v γ-PGA (Na+ form. LM); 8% w/v PEG 20000
**35**	JCSG G12	155	186.93	186.93	59.98	90	90	120	1.42	5.5	3 M NaCl. 0.1M Bis Tris
**36**	PACT B8	155	187.14	187.14	60.17	90	90	120	1.43	6.0	0.2 M Ammonium chloride; 0.1 M MES; 20% w/v PEG 6000
**37**	Morpheus E2	155	187.21	187.21	60.12	90	90	120	1.47	6.5	0.12 M Ethylene Glycols. 0.1 M Buffer System 1. 30%v/v EDO_P8K
**38**	Morpheus D5	155	186.8	186.8	60	90	90	120	1.49	7.5	0.12 M Alcohols. 0.1 M Buffer System2.30%v/v P500MME_P20K
**39**	JCSG F10	155	186.93	186.93	60.21	90	90	120	1.51	7.0	1.1 M Sodium Malonate 0.1 M HEPES. 0.5% Jeffamine
**40**	Morpheus A10	155	186.68	186.68	59.8	90	90	120	1.54	8.5	0.06 M Divalents. 0.1M Buffer system3. 30%v/v EDO_P8K
**41**	PGA E12	155	187.3	187.3	60.27	90	90	120	1.54	6.5	0.1 M Ammonium sulfate; 0.3 M Sodium formate; 0.1M Sodium cacodylate; 3% w/v γ-PGA (Na+ form. LM); 10% w/v PEG 2000 MME
**42**	Morpheus A1	155	187.21	187.21	60.28	90	90	120	1.57	6.5	0.06 M Divalents. 0.1 M Buffer system1. 30%v/v P500MME_P20K
**43**	Morpheus A2	155	187.43	187.43	60.32	90	90	120	1.58	6.5	0.06 M Divalents. 0.1 M Buffer System 1. 30%v/v EDO_P8K
**44**	PACT B3	155	187.2	187.2	60.22	90	90	120	1.58	6.0	0.1 M MIB; 25% w/v PEG 1500
**45**	PGA B10	155	187.21	187.21	60.35	90	90	120	1.62	5.0	0.2 M Potassium bromide; 0.2M Potassium thiocyanate; 0.1 M Sodium acetate; 3% w/v γ-PGA (Na+ form. LM); 5% w/v PEG 4000

**46**	Morpheus A3	155	187.16	187.16	60.07	90	90	120	1.67	6.5	0.06 M Divalents. 0.1M Buffer System 1. 30% GOL_P4K
**47**	Morpheus H8	155	186.56	186.56	59.71	90	90	120	1.69	7.5	0.1 M Amino acids. 0.1 M Buffer system 2. 37.50% MPD_P1K_P3350
**48**	Morpheus B4	155	186.41	186.41	59.67	90	90	120	1.7	6.5	0.09 M Halogens. 0.1 M buffer System1. 37.5%v/v (25% v/v MPD; 25% PEG 1000; 25% w/v PEG 3350)
**49**	JCSG F7	155	189.51	189.51	60.92	90	90	120	1.73	7.0	0.8 M Succinic acid
**50**	PACT H8	155	186.71	186.71	59.85	90	90	120	1.75	8.5	0.2 M Sodium sulfate; 0.1 M Bis-Tris propane; 20% w/v PEG 3350
**51**	Morph3 F6	173	153.76	153.76	39.09	90	90	120	1.85	7.5	0.6% antibiotics mix; 0.1 M Buffer System 2; 50% precipitant mix2
**52**	MIDAS B6	155	185.96	185.96	60.78	90	90	120	1.89	7.5	1.5% Vitamins Mix. 0.1 M Buffer System 2. 50% Precipitant Mix 2
**53**	JCSG E8	155	186.88	186.88	60.59	90	90	120	1.96	4.5	1 M Ammonium phosphate dibasic. 0.1M Sodium Acetate
**54**	PACT E3	173	152.91	152.91	39.28	90	90	120	2.1		0.2 M Sodium Iodide; 20% w/v PEG 3350
**55**	MIDAS B1	173	152.07	152.07	39.12	90	90	120	2.11	5.5	0.2 M Sodium chloride; 0.1M MES; 20% v/v Jeffamine D2000. 10%v/v jeffamine M-2005
**56**	Morph3 G1	18	118.36	98.23	104.6	90	90	90	2.16	6.5	1.2% Cholic Acid derivatives mix. 0.1M Buffer System1. 50% precipitant Mix1
**57**	PGA B4	155	180.83	180.83	57.61	90	90	120	2.51	5.0	0.1 M Sodium acetate; 5% w/v γ-PGA (Na+ form. LM); 8% w/v PEG 20000
**58**	PGA B8	5	113.79	179.79	58.86	90	122	90	3	5.0	0.2 M Sodium bromide. 0.2 M Potassium thiocyanate. 0.1M Sodium acetate; 3% w/v γ-PGA (Na+ form. LM); 10% w/v PEG 2000MME

Table S4 Structural comparison of all superposed protein models.

The RMSDs are plotted against the different models and calculated on the bases 4958 atoms.

Structural C_α-rmsd (Å)	$P6_3$_large	$P6_3$_small	R32	$P2_12_12$
$P6_3$_large	--	0.663	0.632	0.547
$P6_3$_small	0.663	--	1.129	1.044
R32	0.632	1.129	--	0.326
$P2_12_12$	0.547	1.044	0.326	--
